# Potential for reducing immobility times of a mobility monitor in-bed sensor system – a stepped-wedge cluster-randomised trial

**DOI:** 10.1186/s12912-023-01658-2

**Published:** 2023-12-16

**Authors:** Sven Ziegler, Claudia Schmoor, Lili M. Schöler, Sam Schepputat, Eyere Takem, Birgit Grotejohann, Inga Steinbrenner, Johanna Feuchtinger

**Affiliations:** 1 Center of Implementing Nursing Care Innovations Freiburg, Nursing Direction, Medical Center, University of Freiburg, Freiburg, Germany; 2 Clinical Trials Unit, Faculty of Medicine and Medical Center, University of Freiburg, Freiburg, Germany; 3 Institute of Genetic Epidemiology, Faculty of Medicine and Medical Center, University of Freiburg, Freiburg, Germany

**Keywords:** Digital technology, In-bed sensor, Mobility monitoring, Nursing, Pressure Ulcer, Repositioning, Prevention, Stepped-wedge cluster-randomised trial

## Abstract

**Background:**

Pressure ulcer prophylaxis is a central topic in clinical care. Pressure-relieving repositioning is strongly recommended for all pressure-sensitive patients. The Mobility Monitor (MoMo) is a technical device that records a patient’s movements and transmits the data to a monitor. This study investigated the extent to which the MoMo sensor system, which records and visualises patients’ movements in bed, supports nurses in performing pressure-relieving repositioning in neurological and neurosurgical intensive care units (ICU).

**Methods:**

This stepped-wedge cluster-randomised trial involved two clusters: one neurological and one neurosurgical ICU. The study was carried out in two steps over three periods between November 2018 and May 2019, with a two-month interval between each step. At the beginning of the study, we equipped 33 beds across the two ICUs with a MoMo system. Our primary endpoint was the immobility rate, which is defined as the patient’s inactive time in bed exceeding two hours without pressure-relieving movements divided by the time the MoMo was in the bed. The immobility rate ranges from 0 to below 1, with higher values indicating lower mobility. Secondary endpoints were the rate of new pressure ulcers and the rate of relevant pressure-relieving repositionings. Relevant repositionings are defined as the number of repositionings identified by the MoMo as a pressure-relieving repositioning divided by the total number of repositionings,

**Results:**

808 patients were included in the study, of whom 403 were in the control group and 405 were in the intervention group.

The mean immobility rate was 0.171 during the control phase and 0.144 during the intervention phase. The estimated intervention effect was -0.0018 (95% confidence interval [-0.0471, 0.0436], *p*=0.94). The number of new pressure ulcers was 5/405 in the intervention phase and 15/403 in the control phase. We noted a small difference in the mean rate of relevant repositioningswith an estimated intervention effect of 0.046 (95% confidence interval [-0.018, 0.110], *p*=0.16).

**Conclusion:**

Our results are insufficient to recommend the standardised use of mobility monitors in neurological or neurosurgical ICUs.

**Clinical trial registration:**

The primary analysis was prespecified and the trial was registered in the German Clinical Trials Register (DRKS) under the reference number DRKS00015492 (31/10/2018).

## Introduction

### Pressure ulcers

Pressure ulcers are defined as “… localized damage to the skin and underlying soft tissue usually over a bony prominence or related to a medical or other device” [1]. They continue to be associated with profound suffering for the patients affected, additional work for the nursing staff and high costs for the health care system [2]. Pressure ulcer prophylaxis is thus a central topic in clinical care. The development of pressure ulcers is a highly complex and individual process in which pressure and individual pressure tolerance, individual tissue reactions, shear forces, the autonomous nervous system (and its anomalies), and the tissue’s recovery capacity are important factors [3–5]. In 2014, the European Pressure Ulcer Advisory Panel (EPUAP), National Pressure Ulcer Advisory Panel (NPUAP) and Pan Pacific Pressure Injury Alliance (PPPIA) paid particular attention to the prevention of pressure ulcers in critically ill patients in their guideline “Prevention and Treatment of Pressure Ulcers”. Pressure-relieving repositioning is strongly recommended for all pressure-sensitive patients (strength of evidence = A; strength of recommendation = strong positive recommendation). The frequency of repositioning depends on each patient’s situation and their tissue’s reaction to pressure [6]. In Germany’s “National Expert Standard on Pressure Ulcer Prevention in Nursing”, which was updated in 2017 by the German Network for Quality Development in Nursing, significant importance is attached to changing positions and relieving the pressure on tissue [7]. However, studies have so far yielded inconsistent data regarding the intervals at which such repositionings should take place [8]. The only significant evidence on the frequency of changing positions suggests that a change in position helps prevent the development of pressure ulcers in general, regardless of the time interval [9].

Nevertheless, the practice of consistently changing positions every two hours has long been recognised as an effective measure for relieving tissue pressure and preventing pressure ulcers [10]. The literature reports a wide range of compliance (38–54%) with the recommended “two-hour change in position” [10–13]. Due to methodological differences in study designs, the reported numbers are not readily comparable. Patients in neurological and neurosurgical intensive care units (ICU) tend to be subject to movement restrictions, and are thus especially susceptible to pressure ulcers [13, 14]. Suboptimal nurse-to-patient ratios [15], difficulties in monitoring patient positioning and ineffective reminders regarding positioning [16] are discussed in the literature as possible obstacles to following the recommended repositioning frequency.

Technical aids can assist in detecting a patient’s movements in bed, facilitating the monitoring of repositioning by employing suitable reminder and alarm systems. The Mobility Monitor (MoMo) is produced by Swiss company compliant concept AG. The MoMo bed sensor is a technical device that records a patient’s movements and transmits the data to a monitor. The MoMo provides staff with real-time information on the frequency of movements and the patient’s pressure-relieving versus non-pressure-relieving movements (micro-movements). A traffic light function on the monitor indicates when a specific time limit without pressure-relieving movement has been reached. The MoMo promises to support caregivers in enabling precisely timed pressure-relieving repositioning that is adapted to individual movement patterns [17]. The MoMo consists of a sensor mat positioned centrally under the patient’s mattress, a control unit attached to the bed and a software-based monitoring unit located on the nurses’ ward. The system is able to distinguish between micro-movements and pressure-relieving changes in the patient’s position. Health care staff can press a button 10 min before or after they initiate a repositioning to assign it as a health care staff-initiated movement. This allows the control unit to differentiate between a health care staff-initiated movement and a patient independently repositioning themselves. A traffic light system informs nursing staff about a patient’s pressure-relieving movements. If the patient has made enough pressure-relieving position changes within a specific period (e.g. two, three or four hours), the light will be green. If no pressure-relieving repositioning has occurred half an hour before the end of the designated period, the light will turn yellow. And if no pressure-relieving repositioning has occurred at the end of the designated period, the light will turn red.

The MoMo has been tested in nursing homes, and in non-ICU inpatient medical and surgical units [18–21].

The present project was carried out to gain knowledge about the MoMo’s use in neurological and neurosurgical ICUs. We assumed that using a MoMo in neurological and neurosurgical ICUs would lead to more frequent and timelier pressure-relieving repositioning of patients, thereby reducing the incidence of pressure ulcers. The perspective of the health care staff was examined through a formative evaluation conducted as part of an accompanying project [22].

## Methods

### Aim

The aim of this study was to investigate the influence of an in-bed mobility monitor on (1) the immobility rate, (2) the incidence of newly developed pressure ulcers, and (3) the rate of relevant pressure-relieving repositionings initiated by health care staff in a neurological and neurosurgical ICU.

The immobility rate is defined as a patient’s inactive time in bed exceeding two hours without pressure-relieving movements divided by the time the MoMo was in the bed. The rate ranges from 0 to below 1, with higher values indicating lower mobility. A score of 0 indicates that the patient’s immobility time during their time in bed never exceeded two hours. The rate of relevant repositionings is defined as the number of repositionings recognised by the MoMo as a pressure-relieving repositioning divided by the total number of repositionings. Health care staff press a button on the MoMo to identify a movement as a health care staff-initiated repositioning. We refer to the movements initiated by health care staff to prevent pressure ulcers as “relevant repositionings” if the MoMo considered the movements sufficiently tissue pressure relieving. The rate of relevant repositionings ranges from 0 to 1, with a higher value indicating more relevant repositionings.

### Design and setting

**Fig. 1:**
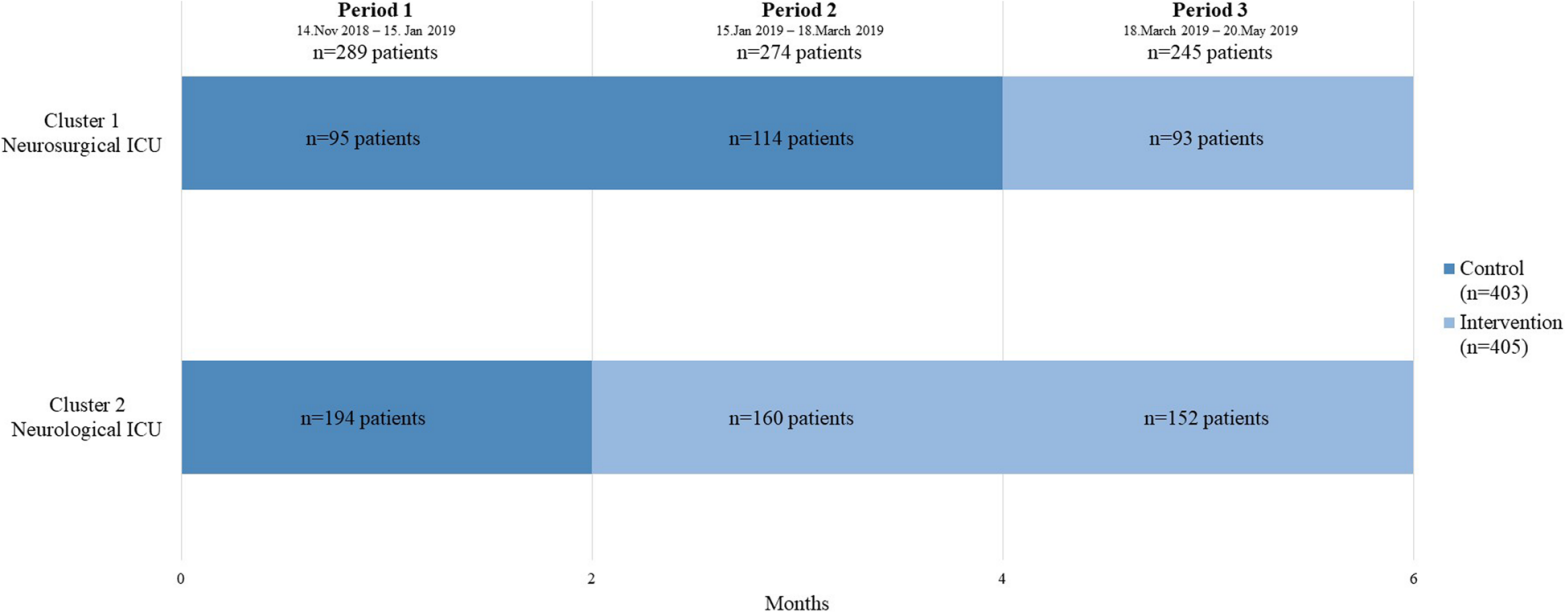
Clusters and allocation to study period

This project was carried out using a cross-sectional stepped-wedge design (Dreischulte et al., 2013; Hemming & Taljaard, 2016) with two steps over three periods and two clusters (Fig. 1). The two clusters comprised one ICU each, namely one neurological and one neurosurgical ICU of the Medical Center – University of Freiburg in Germany. In contrast to a classic cluster-randomised trial, the stepped-wedge design offers the advantage of a comparatively small design effect, even with a limited number of clusters. Furthermore, all ICUs can benefit from the intervention. Since two ICUs took part in this trial, there were three periods. First a control period which included both ICUs, and then two periods in which one ICU transitioned to the intervention phase per step. Since the trial was designed to last six months, the steps were taken after two and after four months. At the end of the first period, the ICUs were randomised, with one ICU remaining in the control phase and one switching to the intervention phase. This process was performed by opening a randomisation envelope at each ICU. To guarantee concealment of the randomisation, the envelopes were centrally prepared by the Clinical Trials Unit at the beginning of the study using the random number generator of the function uniform available in SAS version 9.2. The neurosurgical ICU had 15 beds and an average 3 to 1 nurse-to-patient ratio, with 26 full-time equivalent (FTE) registered nurses (RN) and 7 FTE nurse aids (NA) working in the ICU. The main diagnoses in the neurosurgical ICU were intracranial or intracerebral bleedings and spinal diseases. The neurological ICU had 16 beds and an average 3 to 1 nurse-to-patient ratio with 29 FTE RNs and 3 FTE NAs. The main diagnoses in the neurological ICU were strokes and intracranial bleedings. On both ICUs, all patients receive physiotherapy and nurses are supported by physiotherapists in carrying out mobilisation activities. The mortality rates of the ICUs were 0.07% for the neurosurgical ICU and 0.05% for the neurological ICU. These contextual factors remained stable throughout the six-month study period.

### Sample

Because pressure ulcers can develop rather quickly, we included all patients hospitalised in the neurosurgical or neurological ICU who were in a bed with a MoMo, regardless of the duration of their stay. Reasons why patients were in beds without a MoMo and hence excluded from our study were: (1) Some beds could not be equipped with a MoMo, such as air beds with a fixed mattress and bariatric beds; and (2) patients were transferred from another unit while lying in a bed without a MoMo.

### Intervention

At the beginning of the six-month study period, all 33 beds in the two ICUs were equipped with a MoMo. After patients left the ICU, the MoMos were processed by supply assistants or nursing staff and placed back in the free beds ready for the next patient. Staff were instructed in the use of the MoMo by the manufacturer. A training video was also available to staff via the hospital intranet. Staff questions would be clarified during daily visits by members of the study team (study nurse or research assistant).

Both ICUs began with a blinded control phase (i.e. the MoMo recorded the patients’ movements, but nursing staff had no access to the data). After two months, one of the ICUs was randomly chosen to enter the intervention phase in which the nursing staff could see their patients’ movement data using the MoMo. After another two months, the second ICU also entered the intervention phase.

Throughout the entire study, the nurses followed the standard protocol, which has been in place since 1992, and has undergone frequent review and adaptation based on the latest evidence. All nurses receive regular mandatory training on the content and use of the standard protocol. Pressure ulcers are defined as “… localized damage to the skin and underlying soft tissue usually over a bony prominence, or related to a medical or other device. The injury can present as intact skin or an open ulcer and may be painful. The injury occurs as a result of intense and/or prolonged pressure or pressure in combination with shear” [1]. The standard protocol mandates a skin assessment within the first eight hours following admittance to the unit. Additionally, it advises macro movements to be conducted within four hours. All beds are constantly equipped with pressure-distributing mattresses. An advanced practice nurse (APN) receives an automated report of all documented pressure ulcers with a description of the type and the localisation. The APN then evaluates the documentation, and if necessary makes an additional assessment of the pressure ulcer, provides feedback to the nurses and makes timely corrections to the documentation. The study team presumed that the nurses would adhere to the standard protocol. The standard protocol did not change before or during the study period. The use of the MoMo was in addition to the standard protocol and was not integrated into it.

### Data collection

Data was collected between 14 November 2018 and 20 May 2019. Only the first admission of each patient was included in our primary analysis. No follow-up examination took place after each patient’s stay in a bed equipped with the MoMo. Data on mobility and repositioning was obtained from the MoMo records for the intervention and control groups. Patient data, as well as data on pressure ulcers existing at admission and newly occurring during the study, was taken from clinical records by scientific members of the study team and entered into the data management system secuTrial®. Neither the study team nor the scientists who analysed the data were blinded. The skin assessment and documentation followed the hospital’s standard protocol.

### Statistics

Descriptive analyses were performed by summarising continuous data using the arithmetic mean, standard deviation, and the number of complete and missing observations. Categorical data was summarised by the total and relative frequencies of patients in each category. All analyses were performed using SAS 9.4. All patients hospitalised in the neurosurgical or neurological ICU who were in a bed equipped with a MoMo were included in the analysis, as such the analysis followed an intention-to-treat approach. The analysis was restricted to the first admission of all patients. An analysis of all ICU admissions within the study (i.e. including the subsequent admissions of patients who were admitted to the ICU more than once) was conducted as a sensitivity analysis. The primary endpoint was analysed using a mixed linear model, including the ICU as a random effect, and fixed effects for the intervention, the study period and calendar time (PROC MIXED). Calendar time was defined for each patient as the number of days between the start of the study and the patient’s admission to the ICU, and was included in the model to account for time effects. Analyses of the secondary endpoint, “pressure-ulcer incidence”, were purely descriptive and evaluated using incidence density rates per 10 days in bed. The third endpoint, the “rate of relevant pressure-relieving repositionings initiated by staff”, was analysed using a mixed linear model, with the same covariates as for the primary endpoint, and interpreted in a descriptive sense (PROC MIXED).

Power calculations were based on the primary endpoint (i.e. inactive time in bed) proportional to the time the MoMo was in the bed. In the literature, a proportion of 46–62% immobility time (greater than two hours) was reported [10–13]. Due to the intense and longstanding efforts of the nurses to reduce inactive time in bed, we assumed a lower immobility rate than reported in the literature. Additionally, the numbers reported in the literature have to be interpreted with caution due to methodological differences. For our study, we assumed an immobility rate of 0.30 with a standard deviation of 0.15 during the control phase, based on data from a pilot study [23] and expert opinion. Internal data showed that each ICU treated approximately 1,500 patients per year, resulting in the treatment of a total of 1,500 patients during the six-month study period (i.e. 250 patients per ICU per period). We assumed that 20% of patients would not fulfil the inclusion criteria and that no data would be recordable for 10% of eligible patients, resulting in an estimated 1,080 patients (72%) available for analysis. The design effect in a stepped-wedge design with two steps, 250 patients per cluster per period and an assumed intraclass correlation of 0.1 equals 4.02 [24]. This is the factor by which the available sample size had to be divided to get the effective sample size for achieving the same power as a study with an unclustered design. As such, the effective sample size was expected to be 270 for this study. Therefore, with a two-sided significance level of 5%, this study had a power of 80.9% to detect a difference between interventions, assuming a real difference of 0.052 (nQuery Advisor 7.0).

### Ethical considerations

During the intervention phase, we cannot rule out that the MoMo devices convinced staff to remain inactive, when preventive action was needed. This contrasts with the expected advantages of precise positioning and of avoiding too few or excessive repositionings to prevent pressure-related tissue damage. In the control phase, care followed the accepted standard procedure while the study was conducted. Due to the MoMo’s design, the MoMo did not interfere with the patients’ comfort while lying in bed. The monitoring and recording of movement patterns were limited to micro and pressure-relieving movements, as well as movements initiated, supported and made by health care staff. This meant that only information relevant to nursing care was collected and stored.

The primary analysis was prespecified and the trial was registered in the German Clinical Trials Register (DRKS) under reference number DRKS00015492 (31/10/2018). The study was submitted to and approved by our local university ethics committee (see DRKS Registration) on 31 October 2018.

## Results

### Enrolment

During the study period, we had a total of 1,279 admissions to the two ICUs. Of those, 862 (67%) admissions had a MoMo in the bed, corresponding to a total of 808 patients. As only the first admission of each patient was included in our primary analysis, we excluded the subsequent 54 admissions of 41 patients who were admitted to the ICU more than once (31 patients were admitted twice, eight patients three times, one four times and one five times). We carried out a sensitivity analysis of all 862 admissions, which showed similar results. Of the 808 patients, 403 were enrolled in the control phase and 405 were enrolled in the intervention phase (Fig. 1).

### Demographics and length of stay (with a MoMo)

Out of the 808 patients, 370 were female and 438 were male. In the neurosurgical ICU, the proportion of male patients was approximately 10% higher than that of females; in the neurological ICU, the distribution of genders was almost equal. The percentage of male and female patients differed only slightly between the control phase and intervention phase.

The age distribution varied widely from 16 to 97 years old. The mean age was within a similar range in the two ICUs and during the two study phases (Table 1).

**Table 1:** Patient demographics

Gender*				*n* Total	*n* female / male	*%* female / male
	ICU^a^	Neurosurgical		302	121 / 181	40.1 / 59.9
		Neurological		506	249 / 257	49.2 / 50.8
	Study phase	Control		403	188 / 215	46.7 / 53.3
		Intervention		405	182 / 223	44.9 / 55.1
		**Total**		808	370 / 438	45.8 / 54.2
Age (Years)			Mean	Std^**b**^		
	ICU^a^	Neurosurgical	63.4	16.8		
		Neurological	68.9	16.5		
	Study phase	Control	65.0	17.4		
		Intervention	68.7	16.1		
		**Total**	66.8	16.8		
Time with MoMo (hours)	ICU	Neurosurgical	107:24	167:13		
		Neurological	83:19	98:07		
	Study phase	Control	93:34	140:53		
		Intervention	91:05	115:42		
		**Total**	92:19	128:48		

^*^The hospital information system does not have a classification for non-binary people

^a^Intensive care unit; ^b^Standard deviation

### Outcomes

#### Immobility rate

The immobility rate is defined as the patient’s inactive time in bed exceeding two hours without pressure-relieving movement divided by the length of time the MoMo was in the bed. The immobility rate ranges from 0 to below 1, with higher values indicating lower mobility. The mean immobility rate was 0.171 during the control phase and 0.144 during the intervention phase (Table 2). However, when comparing the control phase to the intervention phase, one needs to consider that the percentage of patients from the neurosurgical ICU was 52% (209/403) in the control phase, but only 23% (93/405) in the intervention phase. Additionally, it should be noted that the neurosurgical ICU had a higher immobility rate than the neurological ICU. In the linear mixed model, which accounted for this imbalance by considering the clustered design, and adjusting for period and calendar time, the estimated intervention effect was − 0.0018 (95% confidence interval [-0.0471, 0.0436], *p* = 0.94). Values below zero indicate higher mobility in the intervention group. In the linear mixed model, the intraclass correlation (ICC) was estimated to be 0.105. This fits very well to the assumed ICC of 0.1 used in the sample size calculation. Therefore, the results indicate that the MoMo had no significant effect on the immobility rate.

**Table 2:** Immobility rates by ICU, period and study phase

Period	ICU^a^	Study phase	Total	Mean	Std^b^
Period 1	Neurosurgical	Control	95	0.21	0.19
	Neurological		194	0.13	0.14
		**Total**	289	0.16	0.17
Period 2	Neurosurgical	Control	114	0.21	0.17
	Neurological	Intervention	160	0.13	0.13
		**Total**	274	0.16	0.15
Period 3	Neurosurgical	Intervention	93	0.20	0.19
	Neurological		152	0.12	0.12
		**Total**	245	0.15	0.16
All periods	Neurosurgical	All study phases	302	0.20	0.18
	Neurological		506	0.13	0.14
		**Total**	808	0.16	0.16
All periods	All ICUs	Control	403	0.17	0.17
		Intervention	405	0.14	0.15
		**Total**	808	0.16	0.16

^a^Intensive care unit; ^b^Standard deviation;

#### Pressure ulcers

A pressure ulcer was observed upon admission in 30 patients (15 in both the neurological and neurosurgical ICUs; 19 in the control phase and 11 in the intervention phase). The number of newly occurring pressure ulcers during the intervention phase (5/405 (1.2%)) was lower than during the control phase (15/403 (3.7%)) (Table 3). All pressure ulcers were rated as grade 1 or 2, using the EPUAP categorisation. The incidence density rate takes into account that the time with a MoMo and thus the time at risk of developing a pressure ulcer was not identical among patients. The incidence density rate was consistently higher in the neurosurgical ICU compared to the neurological ICU (Table 3). Within each ICU and in total, the incidence density rate was lower during the intervention phase (0.022 per 10 days the patient spent in bed) than during the control phase (0.070 per 10 days the patient spent in bed).

**Table 3:** Cumulative time at risk, number of new pressure ulcers and corresponding density rates per 10 days the patient spent in bed

Period	ICU^a^	Study phase	N° of patients	N° of patient-days at risk	N° of new pressure ulcers	Incidence density rate (per 10 days)
Period 1	Neurosurgical	Control	95	654	9	0.138
	Neurological		194	933	2	0.021
Period 2	Neurosurgical	Control	114	565	4	0.071
	Neurological	Intervention	160	845	1	0.012
Period 3	Neurosurgical	Intervention	93	530	3	0.057
	Neurological		152	831	1	0.012
All periods	Neurosurgical	Control	209	1219	13	0.107
		Intervention	93	530	3	0.057
	Neurological	Control	194	933	2	0.021
		Intervention	312	1676	2	0.012
All periods	All ICUs	Control	403	2152	15	0.070
		Intervention	405	2206	5	0.022

^a^Intensive care unit

#### Rate of relevant staff-initiated or supported repositionings

The rate of relevant repositionings ranges from 0 to 1, with a higher value indicating more relevant repositionings. The mean rate of relevant repositionings was 0.70 during the control phase and 0.68 during the intervention phase (Table 4). In the linear mixed model, accounting for the clustered design, and adjusting for period and calendar time, the estimated difference of rates between intervention and control phase was 0.046 (95% confidence interval [-0.018, 0.110], *p* = 0.16). Thus, the rate of relevant repositionings was slightly increased by the intervention, but the 95% confidence interval includes the value of zero. As no repositionings were recorded for 108 patients, no relevant repositionings were also recorded for these patients.

**Table 4:** Rate of relevant repositionings in relation to all documented repositionings

Study phase	Total	No repositioning	Total valid	Mean	Std^a^
Control	403	47	356	0.70	0.24
Intervention	405	61	344	0.68	0.26
**Total**	808	108	700	0.69	0.25

^a^Standard deviation;

## Discussion

In this stepped wedge, cluster randomized trial on two ICUs including 808 patients we observed no relevant effects of the MoMo on any of our measured outcomes.

As one of the reasons discussed in the literature for prolonged immobility time is ineffective reminders regarding positioning, the objective of this study was to investigate whether a MoMo system could reduce patients’ immobile time in bed, reduce the incidence of pressure ulcers and increase the rate of relevant repositionings initiated by health care staff. To investigate these questions, we equipped all available beds in two neurosurgical and neurological ICUs in Germany with a MoMo. The ICUs were then assigned to one of two phases (control or intervention) of the study using a stepped-wedge design.

Our study’s primary outcome, namely the immobility rate, showed no significant difference between the intervention and control phases (intervention effect − 0.0018 (95% confidence interval [-0.0471, 0.0436], *p* = 0.94)). However, we observed a relatively low mean immobility rate (0.171 during the control phase and 0.144 during the intervention phase). This suggests that the standard protocol for pressure ulcer prevention was well implemented. Furthermore, the mean immobility rate is much lower than described in the literature [10, 11, 13], which was the basis for our power calculation. Even assuming 0.30, which was already below the values described in the literature, as a starting point for the power calculation during the planning phase of the study proved to be too high. The immobility rate during the control phase was consistently lower than 0.30. Given such a low immobility rate, we would have had to enrol many more patients in the study to have sufficient power to examine the effect of the MoMo.

Regarding our secondary endpoints, we observed a lower incidence of new pressure ulcers during the intervention than during the control phase (*n* = 5 vs. *n* = 15). The calculations of the incidence density rate revealed that it was lower during the intervention phase than the control phase. However, these differences must be interpreted cautiously due to the low number of pressure ulcers. As we did not investigate whether the nurses strictly followed the standard protocol for pressure ulcer prevention, we cannot rule out the possibility that some pressure ulcers were not detected. Compared to the reported numbers from other neurological ICUs and ICUs in Germany, the incidence of pressure ulcers was low (2.5% vs. 12–14.9%) [25, 26]. Nevertheless, the fact that we found only grade 1 or 2 pressure ulcers underpins our assumption that the nurses followed the standard protocol. If only higher-grade pressure ulcers had been observed, one could assume that there might be an under-detection of pressure ulcers. Additionally, the low immobility rates we found suggest that the nurses followed the standard protocol. Hence, the low number of pressure ulcers might be attributable to the intensive efforts made by nursing staff in both ICUs to improve pressure ulcer prophylaxis years before the start of this study. Another reason for the low number of pressure ulcers could be that they occurred after discharge from the ICU. Since we did not conduct any follow-up, we cannot rule this out. Furthermore, the nurses’ awareness of being involved in a study and their knowledge that a monitoring system was continuously recording their positioning habits could have contributed to a reduction in pressure ulcer incidence and immobility rates. The primary analysis was restricted to the first admission of all patients. Three additional pressure ulcers occurred during subsequent admissions (two in the intervention phase and one in the control phase). We carried out a sensitivity analysis of all 862 admissions, which showed similar results.

The efficiency of repositioning also failed to reveal any difference between the control and the intervention phases. Nevertheless, approximately 70% of the repositionings, which were performed by health care staff to initiate pressure ulcer prevention, were recognised as sufficiently pressure relieving. The remaining 30% of repositionings, which failed in their intention, should be analysed and discussed internally, as they are not good for the patient (unnecessary disturbance) or staff (resource scarcity). Additionally, some nurses reported that they sometimes did not press the button within the required timeframe (10 min before or after repositioning a patient) and pressed the button at some other time instead.

An additional aspect of our project includes the formative evaluation of health care staff perspectives [22, 27]. The formative evaluation found that the MoMo is useful in general, but less so in intensive care settings. The reason for this is that health care staff in ICUs already closely monitor their patients and can promptly react to immobility issues. In contrast, the MoMo system is considered significantly more beneficial for use on regular wards, where patients cannot be monitored as frequently and each nurse cares for a larger number of patients. This is particularly true during night shifts. This finding is supported by MoMo usability testing on two regular wards [23].

### Limitations

Several factors that could have influenced the development of pressure ulcers were not measured in our study. For example, individual risk factors, such as the patient’s diagnosis, repositioning restrictions, nutritional status and skin status, were not assessed. Furthermore, we did not collect data about staffing resources, which if included would have increased the reliability of our models. However, due to time constraints and limited resources, we decided not to collect this data.

Another limitation of this study is its monocentric nature, focusing solely on ICUs within two inter-related specialties. Factors such as the size of the ICUs and clinic, as well as the characteristics of country-specific health care systems may also play a significant role. While content and funding considerations led to this approach, it naturally limits the generalisability of our data.

Additionally, the small number of available wards and logistical constraints limited the design choices. For example, an interrupted time series design may be considered for further research.

## Conclusion

Our study findings can serve as a basis for further studies focusing on managing mobility and technical aids. For further investigations, higher proportions of immobility rates should be presumed to conduct the power calculation to demonstrate an effect. Additionally, it would be important to assess whether such technical aids can help to implement individualised positioning schedules, which would be an additional advantage of using such a system. However, based on our results, we cannot currently recommend the standard use of these aids in neurological and neurosurgical ICUs.

## Data Availability

The datasets generated and/or analysed during the current study are not publicly available due to data protection regulations. However, the datasets are available from the corresponding author on reasonable request.

## Abbreviations

ICU: Intensive care unit
MoMo: Mobility Monitor
Std: Standard deviation
IQR: Interquartile range
